# The Herpesvirus Associated Ubiquitin Specific Protease, USP7, Is a Negative Regulator of PML Proteins and PML Nuclear Bodies

**DOI:** 10.1371/journal.pone.0016598

**Published:** 2011-01-31

**Authors:** Feroz Sarkari, Xueqi Wang, Tin Nguyen, Lori Frappier

**Affiliations:** Department of Molecular Genetics, University of Toronto, Toronto, Canada; Queensland Institute of Medical Research, Australia

## Abstract

The PML tumor suppressor is the founding component of the multiprotein nuclear structures known as PML nuclear bodies (PML-NBs), which control several cellular functions including apoptosis and antiviral effects. The ubiquitin specific protease USP7 (also called HAUSP) is known to associate with PML-NBs and to be a tight binding partner of two herpesvirus proteins that disrupt PML NBs. Here we investigated whether USP7 itself regulates PML-NBs. Silencing of USP7 was found to increase the number of PML-NBs, to increase the levels of PML protein and to inhibit PML polyubiquitylation in nasopharyngeal carcinoma cells. This effect of USP7 was independent of p53 as PML loss was observed in p53-null cells. PML-NBs disruption was induced by USP7 overexpression independently of its catalytic activity and was induced by either of the protein interaction domains of USP7, each of which localized to PML-NBs. USP7 also disrupted NBs formed from some single PML isoforms, most notably isoforms I and IV. CK2α and RNF4, which are known regulators of PML, were dispensable for USP7-associated PML-NB disruption. The results are consistent with a novel model of PML regulation where a deubiquitylase disrupts PML-NBs through recruitment of another cellular protein(s) to PML NBs, independently of its catalytic activity.

## Introduction

The promyelocytic leukemia (PML) tumor suppressor protein exists as multiple isoforms most of which associate to form PML nuclear bodies (PML-NBs)[1], [2], [3]. Through associations with many additional proteins, PML-NBs control several cellular functions including p53 activation, apoptosis, senescence, DNA damage repair and innate antiviral responses [4]. Due to these important roles, loss of PML-NBs is associated with cancer development or progression in a variety of solid tumours and disruption of PML function through fusion to RARα plays a causative role in promyelocytic leukaemia [5], [6], [7], [8]. In general loss of PML is not due to changes in PML transcripts [7] but rather occurs at the level of the protein, triggered by post-translational modifications of PML.

PML is subject to several post-translational modifications. PML is phosphorylated by casein kinase-2 (CK2), an event that targets PML for degradation via ubiquitin-dependant and proteasome-mediated pathway [9], [10]. However, the ubiquitin ligase responsible for CK2-induced polyubiquitylation and degradation of PML is not presently known. PML is also modified by the addition of small ubiquitin-like modifiers (SUMO), a requirement for PML-NB formation [4]. PML SUMOylation is increased in response to arsenic treatment, resulting in the recruitment of SUMO-dependant ubiquitin ligase RNF4, which then ubiquitylates PML and commits it to proteasome mediated degradation [11], [12], [13]. Although post-translational modifications are integral to PML-NB functions and maintaining PML levels, they are incompletely understood [5]. In particular, the understanding of processes that govern PML-ubiquitylation is still in its infancy. For instance, while there is evidence for PML de-SUMOylation by SENP-1 and SENP-5 [14], [15], whether PML ubiquitylation is also regulated by deubiquitylating enzymes (DUBs) remains unknown.

Studies with herpesviral proteins have offered another perspective on the regulation of PML levels and functions. The ICP0 protein of herpes simplex virus (HSV), which is an E3 ubiquitin ligase, was shown to disrupt PML-NBs and degrade PML protein by a mechanism that involves polyubiquitylation and proteasome-mediated degradation of PML [16], [17], [18], [19]. These effects require the catalytic RING domain of ICP0 and, at least in some cell backgrounds, correlates with the ability of ICP0 to interact with the ubiqutin specific protease, USP7 [20]. USP7 was originally identified as an interacting partner of ICP0 that partially localized to PML-NBs [21], [22], [23]. Further studies have revealed that USP7 regulates the autoubiquitylating activity and thus the stability of ICP0 [24], [25]. USP7 has also been widely studied as a regulator of the p53 tumor suppressor [26], [27], [28], [29], [30]. USP7 deubiquitylates and stabilizes not only p53, but also its predominant ubiquitin ligase, Mdm2 and its functional regulator MdmX [31]. By stabilizing p53 and its negative regulators, USP7 offers an elegant way to fine tune the levels and the activity of p53.

Using proteomic approaches, we have previously identified an interaction between the Epstein-Barr virus (EBV) protein EBNA1 and USP7 [32]. Unlike ICP0, EBNA1 does not appear to be stabilized by USP7 [32]. However the USP7-EBNA1 interaction has important consequences for the host and the virus alike. In keeping with the role of USP7 in the p53 pathway, EBNA1 can interfere with the stabilization of p53 by USP7 by effectively competing with p53 for its binding site in the USP7 N-terminal domain (NTD) [33], [34]. EBNA1 can also recruit USP7 to EBV sequences that regulate viral gene expression and genome persistence where, in complex with GMP synthetase, USP7 can deubiquitylate histone H2B and affect EBNA1-mediated transcriptional activation [35]. In addition, like ICP0, EBNA1 was recently shown to disrupt PML-NBs by inducing the degradation of the PML protein [36]. This effect of EBNA1 was not seen using an EBNA1 mutant defective in USP7-binding nor was it evident when wildtype EBNA1 was expressed in conjunction with USP7 silencing. The combined results implicate USP7 in the regulation of PML-NBs.

In this study we explored the possibility that USP7 directly regulates PML. Contrary to observations with other targets of USP7, we found that USP7 negatively regulates PML levels and PML-NBs by a mechanism that is independent of its catalytic activity and independent of the previously described PML regulators CK2 and RNF4.

## Results

### USP7 negatively regulates PML-NBs

**Figure 1:**
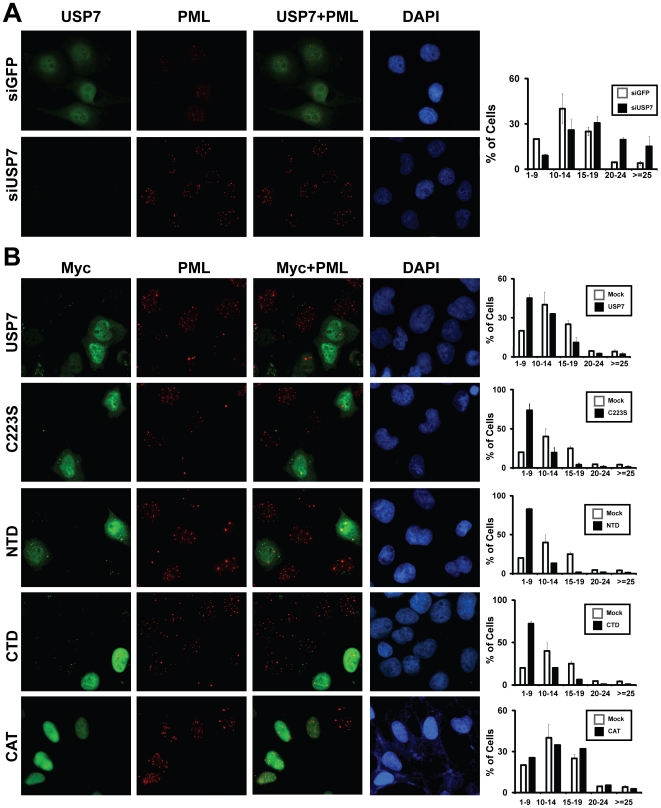
USP7 regulates PML-NBs independent of its ubiquitin cleavage activity. (**A**) CNE2 cells were treated with siRNA against USP7 (siUSP7) or GFP (siGFP) then cells were fixed and stained for PML and endogenous USP7. The number of PML-NBs was counted for a minimum of 50 cells for each sample in three independent experiments. Histogram on the right represents the average distribution of number of PML-NBs in siGFP (white bars) and siUSP7 (black bars) samples, where error bars represent standard deviation from the three independent experiments. Images shown are for siUSP7 samples in which green stained cells serves as an internal control for cells in which USP7 expression has not been silenced. (**B**) CNE2 cells were transiently transfected with 2 µg of a plasmid expressing WT USP7 or USP7 domains as indicated or with the empty plasmid (Mock). 24 hours post-transfection, cells were fixed and stained using PML (red) and c-Myc (green) antibodies. Effect of USP7 overexpression on the number of PML-NBs was quantified as in (A), except at least 100 cells were counted for each sample.

In order to investigate the potential role of USP7 in regulating PML-NBs, we treated the EBV-negative nasopharyngeal carcinoma cell line, CNE2, with siRNA for USP7 (siUSP7) or control siRNA for GFP (siGFP). Immunofluorescence imaging of these cells, clearly showed increased PML staining in cells silenced for USP7 expression and quantification of the number of PML-NBs showed that the average number of PML-NBs per cell increased from 13 ± 0.1 in siGFP cells to 18±0.8 in USP7-silenced cells (Figure 1A). The distribution of the number of PML-NBs per cell revealed that USP7-silenced cells had a large increase in the percentage of cells with more than 19 PML-NBs and a decrease in the number of cells with less than 15 PML-NBs (Figure 1A, right panel). Occasionally we were able to find cells in the same field of view that had both USP7-silenced cells and neighbouring cells that continued to express USP7 (Figure S1), allowing for a direct comparison of PML-NBs in silenced and non-silenced cells in the same culture. Such images also showed that PML staining was much stronger in USP7-silenced cells, than in cells in which USP7 expression was still detectable.

We further examined the role of USP7 in regulating PML-NBs, by overexpressing myc-tagged USP7 in CNE2 cells. While the number of PML-NBs greatly varied from cell to cell, overexpression of USP7 led to a reduction in the number of PML-NBs (Figure 1B, top panel). In multiple experiments the average number of PML-NBs decreased from 13±0.12 in untransfected cells to 9±0.9 in cells overexpressing USP7. In addition, for USP7 overexpressing cells, there was a dramatic increase in the percentage of cells with fewer than 10 PML-NBs compared to untransfected cells (Figure 1B, top panel). Together the results show that USP7 has a destabilizing effect on PML-NBs.

### USP7 catalytic activity is dispensable for PML-NB disruption

**Figure 2:**
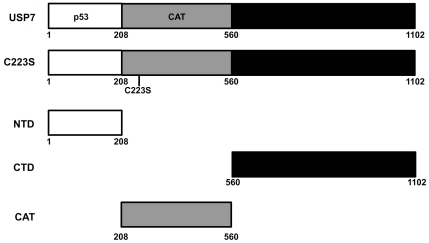
The USP7 proteins used in this study. Pertinent regions of USP7 including, the N-terminal domain (NTD), the C-terminal domain (CTD), the catalytic domain (CAT), p53 binding region and the point mutation (C233S) that abrogates catalytic activity are indicated relative to amino acids numbers (shown below).

The finding that a deubiquitinase destabilized a protein, as opposed to a stabilizing it, was surprising and prompted us to ask whether the catalytic activity of USP7 was required for its effect on PML-NBs. To this end, CNE2 cells were transfected with a construct expressing USP7 with a point mutation in cysteine 223 (C223S) known to be critical for ubiquitin cleavage [28] (Figure 2). Interestingly, expression of C223S reduced the number of PML-NBs to a greater degree than WT USP7 (Figure 1B, 2nd row). The average number of PML-NBs in C223S-positive cells was reduced to 7±1.2 as compared to 13±0.12 in control cells, with a substantially larger percentage of cells containing fewer than 10 PML-NBs than for control cells or even for cells expressing WT USP7. These results indicate that USP7 regulates PML-NBs through a mechanism that is independent of its catalytic activity.

We next examined which USP7 domain(s) was responsible for PML-NB disruption. It has been shown that both the N-terminal (amino acids 1-208; NTD) and C-terminal (amino acids 560-1102; CTD) regions of USP7 form stable structural domains that mediate protein interactions [33] (see Figure 2). Therefore we expressed these domains individually in CNE2 cells (fused to a nuclear localization sequence to ensure nuclear uptake) and examined PML-NB numbers and morphology. Expression of either the NTD or CTD dramatically decreased the number of PML-NBs, comparable in magnitude to C223S (Figure 1B, rows 3 and 4). We also noticed that, in addition to reducing the number of PML-NBs, USP7 overexpression altered their morphology. While there were fewer PML-NBs in cells expressing USP7, C223S, NTD and CTD, the bodies that remained in these cells tended to be larger and stained brighter for PML than those in untransfected cells (Figure 1B). We also expressed the USP7 catalytic domain (208–560) on its own in the CNE2 cells and saw no obvious changes in the size or number of the PML NBs relative to untransfected cells (Figure 1B, bottom row). Taken together these results indicate that USP7 negatively regulates PML-NBs, through both the N- and C-terminal domains and that its catalytic activity is dispensable for this effect.

### USP7 N- and C-terminal domains localize to PML-NBs

**Figure 3:**
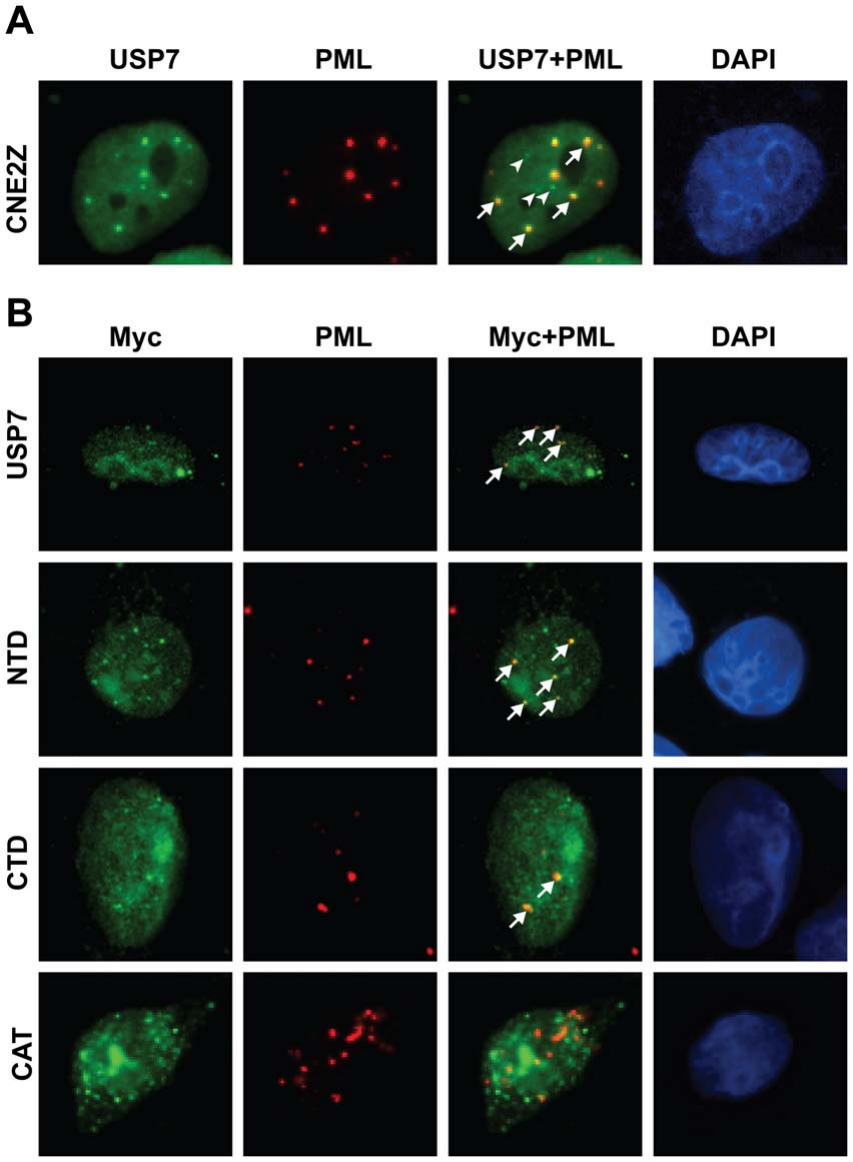
USP7 N- and C-terminal domains associate with PML NBs. (**A**) CNE2 cells were stained for endogenous USP7 and PML. USP7 foci that localize to PML NBs (arrows) or do not localize to PML NBs (arrow heads) are indicated. (**B**) CNE2 cells were transfected with plasmids expressing USP7, the USP NTD, the USP7 CTD or the USP7 catalytic domain (CAT). Cells were harvested 18 hours post-transfection and were extracted with 0.5% Triton X-100 on ice for 4 minutes, prior to fixing and staining with PML and c-Myc antibodies. Arrows identify USP7 foci that localize to PML-NBs.

In most cells USP7 is found throughout the nucleus, often forming discrete foci, some of which associate with PML-NBs (Figure 3A). However how USP7 associates with PML-NBs is not known. To better understand how the USP7 NTD and CTD affect PML-NBs, we asked whether, like WT USP7, they associated with PML-NBs. Immunofluorescence microscopy of ectopically expressed USP7 or USP7 mutants yielded bright pan nuclear staining, making it difficult to determine if the USP7 proteins formed PML-associated foci. To circumvent this problem, we briefly triton-extracted the CNE2 cells to remove excess nucleosolic proteins, soon after transfection with USP7 expression plasmids when PML-NBs are largely intact. The myc-tagged full length USP7 formed foci that closely associated with PML-NBs (Figure 3B, top panel) in a manner similar to endogenous USP7. Additionally, both the USP7 NTD and CTD formed nuclear foci, some of which were closely associated with PML-NBs (Figure 3B, two middle panels). In contrast, we did not see any obvious association of the USP7-CAT domain with PML-NBs (Figure 3B, bottom panel). This suggests that the USP7 domains that disrupt PML-NBs do so through interactions with them. We also noticed that the over-expressed USP7 domains formed some foci that were not associated with PML-NBs, similar to what is observed with endogenous USP7. The nature and function of these foci is not yet known.

### USP7 regulates PML protein levels

**Figure 4:**
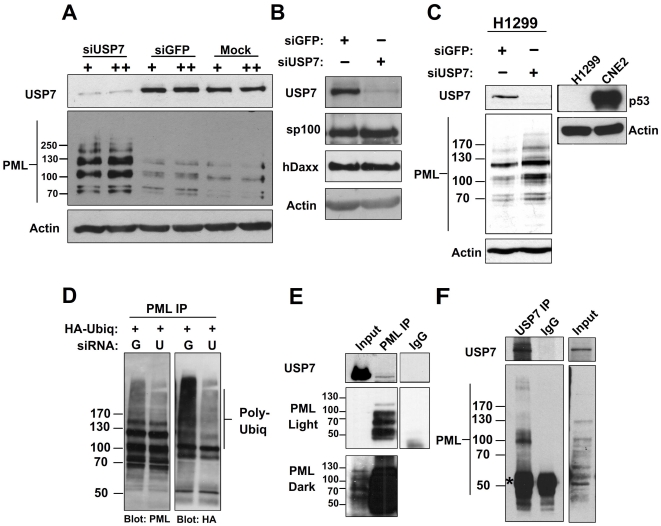
USP7 regulates PML protein levels and physically interacts with PML. (**A–C**) CNE2 cells (A and B) or H1299 cells (C) were subjected to 2 rounds (+) or 3 rounds (++) of transfections with siRNA against USP7 (siUSP7) or GFP (siGFP) or no siRNA (mock). Equal amounts of total cell lysates were analyzed by western blotting using the antibodies indicated. In (C) the blot on the right confirms the lack of p53 expression in H1299 as compared to CNE2 cells. (**D**) CNE2 cells were treated with siRNA against USP7 or GFP then were transfected with a plasmid expressing HA-tagged ubiquitin. PML ubiquitylation was then analyzed by immunoprecipitating endogenous PML and western blotting with HA antibody. (**E and F**) Endogenous PML (**E**) or USP7 (**F**) was immunoprecipitated from CNE2 nuclear extracts using anti-PML or anti-USP7 antibody then western blotted using the reciprocal antibody as indicated. Normal rabbit IgG was used a negative control (IgG) for non-specific immunoprecipitation. ‘Input’ represents 5% of the nuclear lysate used for immunoprecipitation. The band representing IgG is marked by an asterisk.

PML-NBs can be disrupted due to loss of PML proteins or due to failure of the PML proteins to interact to form a NB. These two mechanisms can be distinguished by Western blotting for the PML proteins since only the first mechanism results in decreased levels of PML proteins. To this end, we examined the levels of the PML proteins before and after silencing USP7 in CNE2 cells. Treatment of CNE2 cells with USP7 siRNA efficiently decreased USP7 levels, compared to treatment with siRNA for GFP or transfection reagent alone (Figure 4A, top panel). In SDS-PAGE, PML proteins migrate as a ladder of multiple isoforms representing products of alternatively spliced transcripts and their post-translationally modified versions [2]. Compared to the siGFP or mock treated controls, USP7 silencing led to an increase in most or all isoforms of PML (Figure 4A, middle panel). USP7 silencing specifically affected PML proteins as opposed to other PML-NB components, as the levels of the Sp100 and hDaxx proteins, known to be highly associated with PML-NBs, were largely unaffected by USP7 silencing (Figure 4B).

USP7 can alter levels of the p53 tumor suppressor [37] and p53 can activate PML transcription [38]. The effect of USP7 on p53 levels requires the catalytic activity of USP7, and therefore our finding that catalytically inactive USP7 disrupts PML NBs strongly suggests that this effect is not due to p53 modulation. However, to further verify that the increase in PML levels after USP7 silencing was not due to changes in the stability of p53, we repeated USP7 silencing experiments in p53-negative H1299 cells. Silencing of USP7 caused an increase in PML levels even in the absence of p53 (Figure 4C), indicating that the ability of USP7 to modulate PML levels is independent of p53. Microscopy images of H1299 and Saos2 cells (also p53-negative) after overexpression of USP7 or the C223S mutant also confirmed that USP7 can trigger loss of PML NBs in the absence of p53 and that the effect is independent of ubiquitin cleavage by USP7 (Figure S2).

Since PML protein degradation is mediated by polyubiquitylation, we asked whether USP7 affects the degree of PML ubiquitylation. To this end, CNE2 cells were transfected with a construct expressing HA-tagged ubiquitin along with siRNA against GFP or USP7. Transfected cells were also treated with the proteasome inhibitor MG132 to allow polyubiquitylated proteins to accumulate. Immunoprecipitation of endogenous PML recovered comparable amounts of PML from both siGFP and siUSP7 treated samples (Figure 4D, left panel), however, when the same samples were probed for HA, the level of PML containing HA-ubiquitin was greatly decreased after USP7 silencing (Figure 4D, right panel). Therefore USP7 is required for optimal polyubiquitylation of PML.

### USP7 physically interacts with PML

Whether or not the association of USP7 with PML-NBs involves an interaction with PML proteins is unknown. To test this possibility, we immunoprecipitated endogenous PML isoforms from nuclear extracts and blotted for USP7. This revealed that a fraction of the endogenous USP7 interacted with the PML proteins (Figure 4E). We also conducted the reciprocal experiment in which an antibody against USP7 was used to pull down USP7 (Figure 4F), as indicated by two bands characteristic of USP7 [39]. Western blots for total PML revealed that one PML isoform preferentially coimmunoprecipitated with USP7 (Figure 4F) and its size is consistent with PML IV. These data suggest that USP7 physically interacts with PML-NBs through PML proteins and has a preference for PML IV.

### CK2 is dispensable for USP7 induced PML-NB disruption

**Figure 5:**
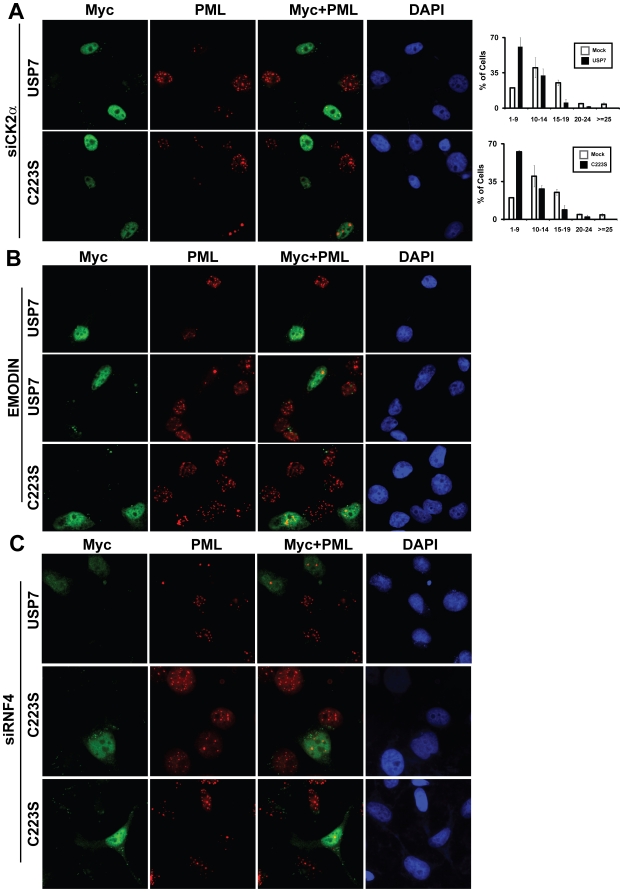
Requirement for CK2α and RNF4 for USP7-induced PML-NB degradation. (**A**) CNE2 cells were transfected with siRNAs against RNF4, USP7, CK2α or GFP and silencing was confirmed by western blotting (see Fig. S3). 24 hours after the last round of siRNA transfections, CNE2 cells silenced for CK2α expression were transfected with a plasmid expressing myc-tagged USP7 or my-tagged C223S (as in Figure 1B). 24 hours later cells were fixed and stained with anti-PML and anti-c-Myc antibodies. PML-NBs was quantified as in Figure 1B. (**B**) CNE2 cells were treated with 50 µg/mL emodin 24 hours prior to transfection with USP7 or C223S expression plasmids. 6 hours post transfection, cells were given a second 24 hr emodin treatment, followed by fixation and immunofluorescence microscopy as in (A). Two different fields of view are shown for USP7 samples to show reproducibility. (**C**) Cells were treated as in (A), except siRNA against RNF4 was used to silence RNF4, prior to USP7 and C223S overexpression. Two different fields of view are included for C223S to show reproducibility.

Scaglioni and colleagues [10] showed that casein kinase 2 (CK2) phosphorylates PML proteins on serine 517 which primes them for polyubiquitylation and proteasome-mediated degradation. Therefore we wondered if the USP7-associated degradation of PML required PML phosphorylation by CK2. We tested this possibility by assaying USP7-associated PML-NB disruption in CNE2 cells deprived of CK2 activity in the following two ways. First, we silenced CK2α using siRNA and then overexpressed either USP7 or C223S in the siRNA treated cells. CK2α expression was efficiently decreased by siRNA treatment and did not affect USP7 levels, as shown in Figure S3 (compare lanes 3 and 4). Consistent with previous reports of a role for CK2 in PML turnover [10], CK2 silencing resulted in increased levels of PML-NBs in general, however depletion of CK2 had no noticeable effect on the ability of USP7 or C223S to disrupt PML-NBs (Figure 5A). We also verified that CK2 was silenced in cells overexpressing USP7 by staining the cells for both CK2 and the myc tag on USP7. As shown in Figure S4, CK2 was depleted in virtually all of the cells including those overexpressing USP7.

To rule out the possibility that residual CK2α remaining after silencing was sufficient to bring about USP7-induced PML-NB disruption, we assessed the effect of USP7 and C223S on PML-NBs in CNE2 cells treated with emodin, an inhibitor of CK2 catalytic activity (Figure 5B). As reported previously [10], emodin treatment on its own increased PML staining. However inhibition of CK2 activity by emodin did not hinder disruption of PML-NBs by either USP7 or C223S (Figure 5B). Note that it was not possible to get accurate counts of PML-NBs in these experiments since emodin treatment results in distortion and/or fusion of PML-NBs such that it is not clear where one body ends and the next begins. However the decrease in PML staining was obvious in all USP7- and C223-expressing cells examined. These observations indicate that USP7 negatively regulates PML-NBs in a manner independent of CK2 phosphorylation of PML.

### USP7 is important for arsenic-induced PML degradation

**Figure 6:**
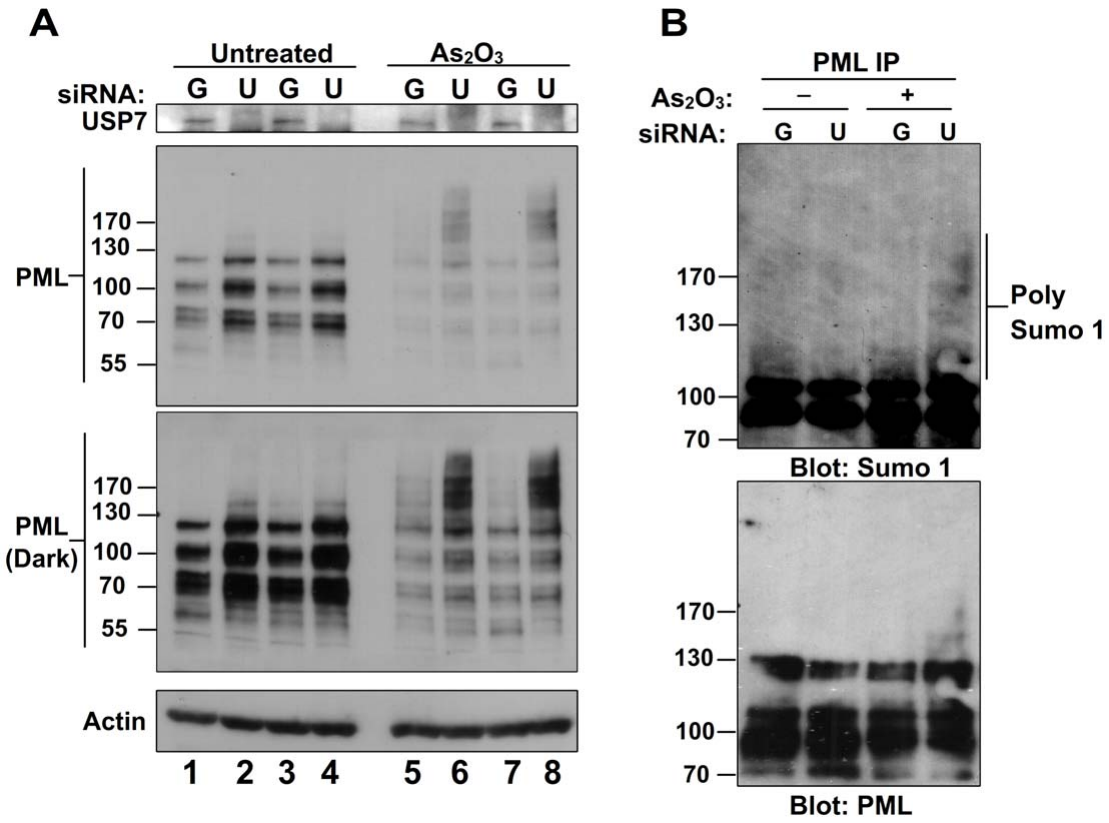
USP7 is important for arsenic-induced PML degradation. (**A**) CNE2 cells were treated with siRNA for USP7 (U) and siRNA for GFP (G) in quadruplicates. 24 hours post-transfection, two samples for each siRNA set were treated with 1 µM As_2_O_3_ for 6 hours then equal amounts of cell extracts were analyzed by western blotting using the antibodies indicated. (**B**) CNE2 cells were treated with siRNA and As_2_O_3_ as in (A). PML was immunoprecipitated from untreated and treated cells and SUMOylated PML was analyzed by western blotting using anti-SUMO1 antibody.

Arsenic is known to induce PML SUMOylation and eventual degradation of PML and is used as a treatment for acute promyelocytic leukemia [40]. To determine whether USP7 is important for arsenic-induced degradation of PML, CNE2 cells were transfected with siRNA against GFP or USP7 in quadruplicates and two of each sample were treated with arsenic trioxide (Figure 6A). As expected, silencing of USP7 resulted in PML stabilization in both replicates in the absence of arsenic trioxide (Figure 6A, compare lanes 1 and 3 with lanes 2 and 4). In siGFP samples, treatment with arsenic trioxide led to degradation of PML as expected (Figure 6A, compare lanes 1 and 3 with 5 and 7). Darker exposures revealed the presence of high molecular weight forms of PML in arsenic treated samples, indicative of PML modified with SUMO and/or ubiquitin. Arsenic treatment of siUSP7 samples did not reduce PML levels to the same degree as in siGFP control cells (Figure 6A, compare lanes 5 and 7 with lanes 6 and 8). While this could be due to the higher starting level of PML after siUSP7 treatment, what is most telling is the striking increase in the proportion of PML migrating as highly modified forms (above the 130 marker) as compared to the unmodified forms (between the 55 and 130 markers). This disproportionate accumulation of higher molecular weight forms of PML, which are normally rapidly degraded, suggests that the degradation of post-translationally modified PML induced by arsenic treatment requires USP7.

The PML high molecular weight forms were further analysed for the presence of SUMO by immunoprecipitation of total PML from each sample and blotting for endogenous SUMO1 (Figure 6B). This confirmed that the slower migrating PML forms evident after USP7 silencing and arsenic treatment (lane 4) were SUMOylated. Note that these SUMO1-modified forms are not detectible in the control siGFP sample, likely because they are degraded readily in presence of USP7. These results indicate that USP7 is dispensible for arsenic-induced SUMOylation of PML but is important for subsequent degradation of these modified isoforms.

### USP7 regulation of PML-NBs is independent of RNF4

The degradation of PML proteins after arsenic treatment involves polyubiquitylation of PML by RNF4 [11], [12], [13]. Therefore we tested whether the USP7-associated disruption of PML-NBs requires RNF4, by treating cells with siRNA against RNF4 prior to overexpressing USP7 or C223S. While siRNF4 treatment significantly decreased RNF4 levels (Figure S3, compare lanes 1 and 3), it did not noticeably affect the ability of USP7 or C223S to disrupt PML-NBs (Figure 5C). Although we could only find a small number (10) of cells that detectably expressed USP7 or C223S after RNF4 silencing, quantification of PML-NBs from these cells revealed that all cells had 5 or fewer PML-NBs with an average of 2.6±1.3 PML-NBs. This is considerably less than in the RNF4 silenced cells without USP7 overexpression which had an average of 17.3±4.0 PML-NBs with none of these cells having 5 or fewer PML-NBs. In addition, USP7 silencing did not affect RNF4 levels (Figure S3, compare lanes 2 and 3) nor did it affect the ability of RNF4 to associate with PML (Figure S5). These findings suggest that USP7 induces degradation of PML by a mechanism that is independent from RNF4-mediated PML degradation.

### USP7 regulates individual PML isoforms

**Figure 7:**
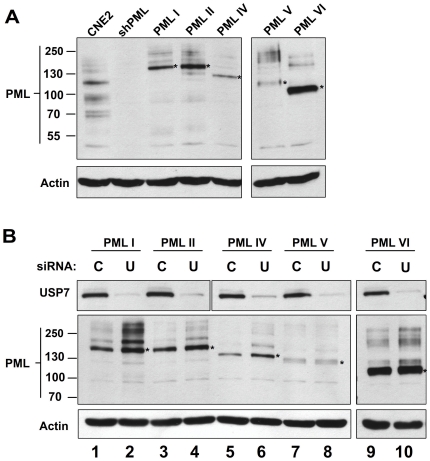
USP7 regulates some individual PML isoforms. (**A**) Endogenous PML expression was silenced in CNE2 cells then silenced cells were engineered to stably express single EYFP-tagged PML isoforms I, II, IV, V and VI. PML content of these cell lines was analysed, relative to parental CNE2 cells, by western blotting equal amount of whole cell lysates and probing with PML antibody recognizing all PML isoforms. The position of each unmodified recombinant PML protein is indicated by an asterisk and the position of molecular weight markers are indicated on the left. A blot for actin is also shown as a loading control. (**B**) Cells described in (A) were transfected with siRNA against USP7 (U) or negative control siRNA (C) then equal amount of cell lysate were analyzed by western blotting using the indicated antibodies.

While silencing of USP7 led to an increase in the levels of all isoforms of endogenous PML, the preferential recovery of one PML form with USP7 in co-IP experiments raised the possibility that PML regulation by USP7 might require a specific PML isoform. To test this possibility, we first silenced endogenous PML in CNE2 cells by stably expressing shRNA against all PML isoforms from a lentivirus as previously described by Everett et al [41]. Expression of shRNA led to depletion of all PML isoforms beyond the limit of detection in CNE2 cells (Figure 7A, compare first two lanes, and Figure S6A). EYFP-tagged PML isoforms resistant to the initial shRNA were then individually and stably expressed in the PML-depleted cells (from the herpes simplex type 1 gD gene promoter) using a second lentivirus [42]. PML isoforms I, II, IV, V and VI were expressed to varying but detectible degrees, while expression of PML III could not be detected and thus was not included in further analysis (Figure 7A and data not shown). In addition, the individual recombinant PML proteins were confirmed by microscopy to form nuclear bodies closely resembling those formed by endogenous PML (Figure S6B). Each of these cell lines was treated with siRNA against USP7 or a negative-control siRNA and effects on the levels of the individual PML proteins examined by western blotting. As shown in Figure 7B, USP7 silencing led to increased levels of PML isoforms I, II and IV, with the largest effects on PML I and IV, while minimal to no effect of USP7 silencing was observed on PML V and VI.

**Figure 8:**
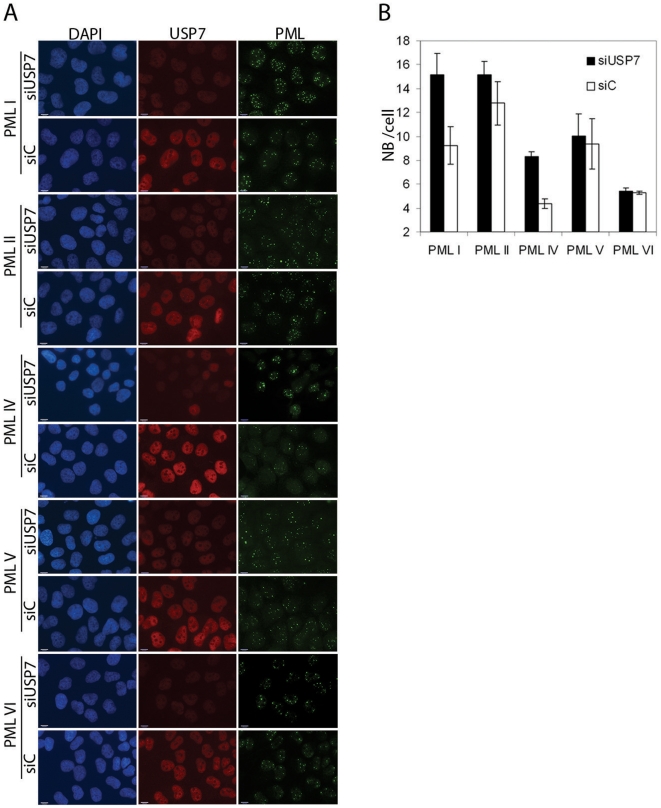
USP7 preferentially affects NBs formed from PML I or IV. (**A**) CNE2 cells expressing the indicated individual PML isoforms were treated with siRNA against USP7 (siUSP7) or negative control siRNA (siC). Cells were then fixed and stained for USP7 and PML and counter stained with DAPI. Images using the same antibody were captured with the same exposure times. (**B**) The number of PML NBs per cell were counted for 100 cells for each of the samples in (A). Average numbers are shown with standard deviations calculated from 3 independent experiments. P values between the control and siUSP7 samples are 0.001 for PML I, 0.1 for PML II, 0.01 for PML IV, 0.2 for PML V and 0.5 for PML VI.

We also examined the nuclear bodies formed by each PML isoform after USP7 and control siRNA treatments and saw a noticeable increase in the number and intensity of PML I and PML IV bodies after silencing USP7, with no obvious effect on the nuclear bodies formed by the other PML isoforms (Figure 8A). Counts of the number of PML bodies per cell showed that the number of nuclear bodies formed varied with different PML isoforms and that only those formed by PML I and PML IV were significantly affected by USP7 silencing, resulting in doubling of the nuclear body number (Figure 8B; p values comparing control and siUSP7 treated samples were 0.001, 0.1, 0.01, 0.2 and 0.5 for PML I, II, IV, V and VI, respectively). These data confirm that USP7 can regulate PML proteins even when expressed from a heterologous promoter, and show that USP7 has a preference for PML isoforms I and IV.

**Figure 9:**
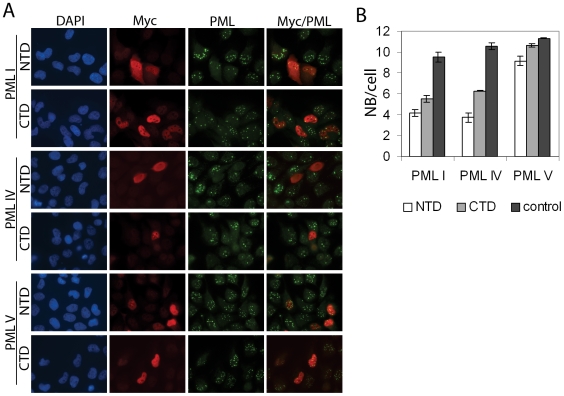
USP7 NTD and CTD both regulate PML I and IV NBs. (**A**) CNE2 cells expressing the indicated individual PML isoforms were transfected with plasmids expressing myc-tagged USP7 NTD or CTD, then were fixed and stained for myc and PML and counter stained with DAPI. (**B**) The number of PML NBs per cell were counted for 100 cells that were expressing or were not expressing (control) the USP7 domain for samples in (A). Average numbers are shown with standard deviations (from 3 independent experiments).

We then investigated whether the USP7 NTD and CTD were sufficient to disrupt the NBs formed from either PML I or PML IV or whether each of these USP7 domains was responsible for regulating one of these PML isoforms. To this end, CNE2 cells expressing only PML I or PML IV (or PML V as a negative control) were transfected with expression plasmids for the USP7 NTD or CTD (fused to a myc tag) and cells expressing these domains were identified using an anti-myc antibody in immunofluorescence microscopy. The PML NBs were visualized by PML staining and counted in cells expressing and not expressing NTD or CTD (Figure 9). Both the NTD and CTD were found to induce pronounced (2–3 fold) loss of PML I and PML IV NBs (p values all between 0.0001 and 0.00027) whereas PML V NBs were much less affected by either the NTD or the CTD. These results confirm the specificity of USP7 and show that the NTD and CTD have similar preferences for PML I and IV NBs.

## Discussion

USP7 is bound by at least two herpesvirus proteins (ICP0 and EBNA1) [22], [32] and has more recently emerged as a key regulator of the p53 tumour suppressor [43] and several other cellular proteins [44], [45], [46], [47]. USP7 can regulate the stability, function and even the sub-cellular localization of its substrates, in each case by virtue of its deubiquitylating activity. Here we showed that USP7 can also function independently from its deubiquitylating activity to negatively regulate the levels of PML proteins and the formation of PML-NBs, by inducing the polyubiquitylation of PML.

A function of USP7 in PML regulation ties in well with its known association with PML-NBs and with its interactions with herpesvirus proteins ICP0 and EBNA1, both of which associate with and disrupt PML-NBs [22], [23], [36]. Indeed the interaction of ICP0 and EBNA1 with USP7 has been found to be important for PML-NB disruption at least in some cell backgrounds [20], [36]. A direct role of USP7 in the regulation of PML levels is consistent with the idea that ICP0 and EBNA1 usurp this role of USP7 to promote degradation of PML proteins and disrupt PML-NBs. In fact our recent studies have shown that EBNA1 independently recruits both USP7 and CK2 to PML NBs (through direct interactions with different EBNA1 sequences) and that recruitment of both USP7 and CK2 is critical for PML disruption [48].

We investigated the mechanism of the association of USP7 with PML-NBs and found that both the N- and C-terminal domains of USP7 were sufficient to mediate this association. These domains also disrupted PML-NBs suggesting that this disruption involved the association between USP7 and PML-NBs. The USP7 NTD is a TRAF domain that is known to bind p53, Mdm2 and MdmX in addition to the viral EBNA1 protein [28], [31], [34], [49]. The C-terminal half of USP7 also appears to have protein interactions as its main function and has been reported to bind FOXO in addition to the viral ICP0 protein [33], [46]. It is not clear that any of these known interactions would account for the targeting to and disruption of PML-NBs by these USP7 domains, and it is likely that additional functionally important interactions with these domains remain to be uncovered.

We also showed that USP7 and PML physically interact in coimmunoprecipitation experiments. Although the PML antibody coimmunoprecipitated only a small amount of endogenous USP7 from nuclear extracts, this is in keeping with previous observations that only a small pool of USP7 forms nuclear foci and that a fraction of these foci colocalize with PML NBs. In reciprocal experiments, USP7 antibody coimmunoprecipitated PML and interestingly, one isoform of PML was preferentially recovered. This suggests that USP7 might be recruited to PML NBs via an interaction with a specific PML isoform, where it can then bring about degradation of all PML isoforms. It is not yet clear if the USP7-PML interaction is direct or mediated by another protein. We have previously shown that EBNA1 also interacts with a single isoform of PML [36] and the size of the isoform that interacts with both EBNA1 and USP7 is consistent with the size of PML IV [50]. This similarity may underscore the importance of USP7 for EBNA1-mediated PML NB disruption.

We examined whether USP7 relied on a specific PML isoform to bring about degradation of the other isoforms using cells expressing single PML isoforms. In keeping with the importance of PML IV for the association of USP7 with PML NBs, we found that USP7 silencing had a significant effect on the number of nuclear bodies formed by PML IV alone but not by PML II, V or VI alone. We also observed significant effects of USP7 on PML I nuclear bodies, suggesting that the PML tail sequence that is shared by PML I and IV but is absent in the other PML isoforms (exon 8a) might be important for USP7-associated PML degradation. Similarly, the levels of PML I and IV were the PML isoforms most affected by USP7 as determined by Western blotting. In addition, the N or C-terminal domains of USP7 that were shown to be sufficient for localization to and disruption of native PML NBs, were also found to be sufficient to disrupt NBs formed by PML I and PML IV (but not PML V). Since the expression of the individual PML isoforms is not driven by the same promoter as for the endogenous PML proteins, these experiments also show that the effects of USP7 siRNA on PML expression is not due to indirect effects from induction of the interferon response. Taken together, the results suggest that preferences of USP7 for specific PML isoforms underlies its ability to trigger loss of PML-NBs and proteins.

**Figure 10:**
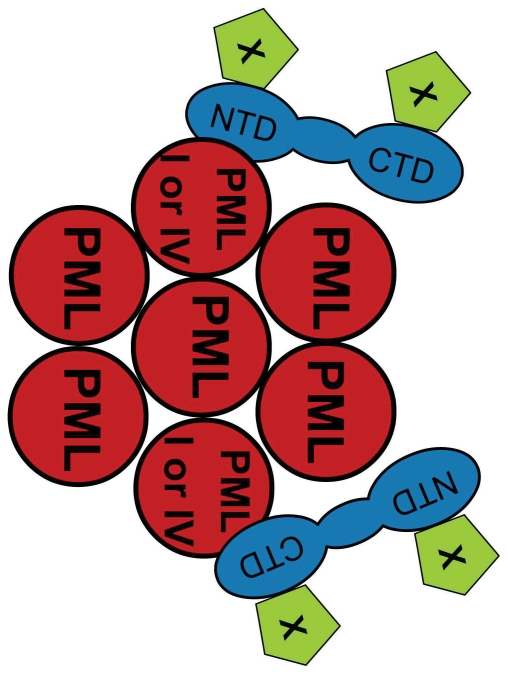
Model of USP7 interactions with PML NBs. Model showing USP7 (blue) interactions through its NTD or CTD with PML proteins I or IV in PML NBs (red). The ability of either the NTD or CTD to disrupt PML NBs suggests that either domain can recruit a PML negative regulator (such as an E3 ubiquitin ligase) to the PML NBs, shown here as protein X (green).

The observations that USP7 promotes PML-ubiquitylation and negatively regulates PML levels were counter intuitive since USP7 generally stabilizes its target proteins by removing their ubiquitin moieties. One possible explanation would be that USP7 stabilizes a ubiquitin ligase that targets PML for ubiquitin-mediated degradation. There is precedent for this possibility as, under some circumstances, USP7 can negatively regulate p53 levels by stabilizing the Mdm2 E3 ligase [26], [28]. However, for PML regulation, this possibility is unlikely given that the USP7 catalytic activity was not required to disrupt PML-NBs. A more likely scenario is that USP7 recruits a negative regulator (see model in Figure 10), for example a ubiquitin ligase, to PML-NBs or that USP7 plays a role in the recruitment of PML to the proteasome for degradation. In support of these possibilities, USP7 has been identified as a component of the proteasome [51], [52] and has so far been reported to interact with two cellular E3 ubiquitin ligases, Mdm2 and MARCH7, in addition to the viral E3 ligase, ICP0 [22], [26], [44]. However Mdm2 does not appear to be required for PML disruption by USP7, as Mdm2 silencing had no noticeable effect on the ability of overexpressed USP7 to induce the loss of PML NBs (data not shown). The ability of USPs to function independently from their catalytic activity is not unique to USP7, as there have been similar reports for USP11 and USP18 (also called UBP43). USP11 upregulates IκB kinase α in a ubiquitin-independent manner [53], while USP18 has been shown to negatively regulate interferon signalling through protein interactions that are independent of its isopeptidase activity [54].

Overall our results point to a novel model of PML regulation in which USP7 triggers degradation of PML by a mechanism that does not require phosphorylation of PML by CK2 or polyubiquitylation by RNF4. Unlike all previously reported USP7 functions, its role in PML regulation does not require the catalytic activity of USP7 but rather is a function of the USP7 protein interaction domains. It is becoming increasingly clear from numerous recent publications that USP7 is an important regulator of many cellular processes. This work further emphasizes the diverse roles of USP7 by showing that the cellular functions of USP7 extend beyond its role as a deubiquitylating enzyme.

## Materials and Methods

### Plasmids

To generate the pCANmycUSP7 plasmid, USP7 cDNA was PCR amplified from the pET3a-USP7 plasmid (a gift from Roger Everett). The amplified fragment was ligated into HindIII and XbaI sites of the pcDNA3.1-derived plasmid, pCANmyc. pCANmycC223S plasmid was generated by QuickChange mutagenesis of pCANmycUSP7 using the following primers: 5′CAGGGAGCGACTTCTTACATGAACAGCCTG3′ and 5′CAGGCTGTTCATGTAAGAAGTCGCTCCCTG3′. USP7 NTD and USP7 CTD fragments were generated by PCR-amplification of the sequences encoding these domains from pCANmycUSP7 using the primers 5′CGCCGCAAGCTTCCGAAAAAAAAAAAACGCAAAGTGATGAACCACCAGCAGCAGC 3′ and 5′ CCGGGATCCTCACTTTGAATCCCACGCAACTCC 3′ for the NTD and 5′CGCCGCAAGCTTCCGAAAAAAAAAAAACGCAAAGTGGAAGCCCATCTCTATATGCAAG 3′ and 5′GCGGGATCCTCAGTTATGGATTTTAATGGCC 3′ for the CTD.

The sequence coding for the SV40 T antigen nuclear localization signal was included in the 5′ primers to generate an in-frame NLS at the N-terminus of each domain. Amplified fragments were ligated into pCMVmyc [55] between HindIII and BamHI sites.

### Cell lines and transfections

CNE2 are EBV-negative nasopharyngeal carcinoma cells (see CNE2Z in [56]). siRNA transfections were performed as described previously [35], except only 50 pmoles of siRNA was used in 6cm dishes. STEALTH siRNA for CK2 and RNF4 was obtained from Invitrogen. 24 hours after siRNA transfections, cells were processed for Western blots or microscopy or moved to larger vessels for plasmid DNA transfections. For overexpression experiments, CNE2 cells (grown on coverslips in a 6cm dish) at 70% confluence were transfected with 2 µg of plasmids expressing myc-tagged USP7 or USP7 domains using lipofectamine 2000 (Invitrogen).

### Immunofluorescence microscopy

Immunofluorescence microscopy was performed as previously described [36] using primary antibodies for USP7 [32], PML (PG-M3, Santa Cruz) or myc (Rabbit A-14 from Santa Cruz or mouse monoclonal 9E10, kindly provided by Dr. Alan Cochrane) and secondary antibodies goat anti-rabbit AlexaFluor 488 and goat anti-mouse Alexafluor 555 from Invitrogen.

### Western Blotting

Cells were lysed in 9 M urea, 5 mM Tris.Cl pH 6.8, followed by brief sonication and microcentrifugation. 30 µg of total protein was subjected to SDS-PAGE and western blots were performed using antibodies against USP7, PML (Bethyl, A301-167A), Sp100 (Santa Cruz, sc-25568), hDaxx (C-20; Santa Cruz, SC-7000,), p53 (DO-1; Santa Cruz, SC-126), CK2α (Abcam, ab10466-50), c-Myc (A-14; Santa Cruz, SC-789), β-Actin (Ab-1; Calbiochem, CP01), SUMO-1 (Zymed), HA (12CA5 monoclonal antibody;[57]) or RNF4 (K7979 [58] kindly provided by Jorma Palvimo) and appropriate HRP-conjugated secondary antibodies for detection by chemiluminescence.

### PML Ubiquitylation Assay

siRNA-treated CNE2 cells were transfected with 5 µg of plasmid expressing HA-tagged ubiquitin (kindly provided by Ron Hay, University of Dundee). 48 hours post-transfection, cells were treated with 10 µM MG132 (Sigma) for 10 hours. Harvested cell pellets were frozen then thawed and boiled in 200 µL of SDS lysis buffer (62.5 mM Tris.Cl pH 6.8, 2% SDS, 10% glycerol,1 mM *N*-ethyl maleimide). Clarified lysates were diluted 5-fold in IP buffer (50 mM Tris.Cl pH 8.0, 150 mM NaCl, 1% NP-40) and precleared with protein A/G sepharose beads (Santa Cruz, SC-2003) and 2 µg normal rabbit IgG (Santa Cruz, SC-2345), prior to overnight incubation with PML antibody (Bethyl, A301-167A). After washing in IP buffer, immunoprecipitates were eluted in loading buffer (60 mM Tris.Cl pH 6.8, 1% SDS, 100 mM DTT, 5% glycerol) prior to western blotting.

### Immunoprecipitation

Nuclei prepared from CNE2 cells by hypotonic lysis were incubated in RIPA buffer (50 mM Tris.Cl pH 8.0, 150 mM NaCl, 1% NP40, 0.1% sodium deoxycholate, 1 mM PMSF and protease inhibitors (P8340, Sigma)) at 4°C for 15 minutes. Nuclear lysates were clarified by centrifugation and precleared with protein A/G sepharose. 1.5 mg of lysate was incubated overnight at 4°C with 2 µg normal rabbit IgG and 2 µg of USP7 (Bethyl, A300-033A) or PML antibodies coupled to ExactaCruz resin (SantaCruz) according to manufacturer's instructions. Immunoprecipitates were immunoblotted as above.

### Cells expressing single PML isoforms

pLKO.shPML1 expressing anti-PML shRNA [41] and pLNGY-PML.I, II, IV, V and VI expressing EYFP-tagged shPML1-resistant PML isoforms were kindly provided by Dr. Roger Everett and are described in detail elsewhere [42]. These plasmids were used to generate lentiviruses as previously described [59]. One ml of filtered culture medium containing the shRNA-lentivirus was added to 1×10^5^ CNE2 cells with polybrene (Sigma) at a final concentration of 8 µg/µl and after 24h was replaced with medium containing 2 µg/ml puromycin. 72 hours later puromycin was removed and cells were cloned by serial dilution and checked for PML expression by immunofluorescence microscopy using antibody against all PML isoforms. A clone with no detectable PML-NBs (designated as CNE2Z-shPML) was further confirmed by Western blotting to lack PML expression and was used to generate cell lines expressing single PML isoforms by incubating 1×10^4^ CNE2Z-shPML cells with 250 µl culture supernatant containing lentivirus encoding a shRNA-resistant PML isoform. Cells containing the second lentivirus were selected in 1 mg/ml G418 (GIBCO) for 7 days, then cloned by serial dilution. Fluorescence microscopy was performed for the EYFP tag to confirm that all cells expressed NBs containing EYFP-PML. Western blots were also performed on 50 µg of total cell extract using anti-PML antibody to confirm the expression of the PML isoform. USP7 silencing experiments were performed in the single PML isoform cells as described above except that cells received 3 rounds of siRNA treatments using AllStars Negative Control siRNA (Qiagen) as a negative control (instead of siGFP), and 40 µg of cell extract was analyzed in the western blots. The microscopy experiments in Figure 8 and 9 were performed as above except that the secondary antibodies goat anti-rabbit AlexaFluor 555 and goat anti-mouse AlexaFluor 488 were used to detect USP7 and PML, respectively.

## Supporting Information

Figure S1 USP7 silencing increases PML staining. CNE2 cells were transfected with siRNA against USP7 and stained for USP7 and PML. An image is shown where a few unsilenced cells remain (green) for direct comparison to the neighbouring cells that are silenced for USP7 expression.
(TIF)

Figure S2 USP7 and C223S overexpression disrupts PML NBs in H1299 and Saos2 cells. Saos 2 (A) or H1299 (B) cells were transfected with a plasmid expressing myc-tagged USP7 or the catalytically inactive USP7 mutant C223S. Cells were fixed and stained for myc and PML as in Figure 1B.
(TIF)

Figure S3 Western blots confirming USP7, RNF4 and CK2α silencing. CNE2 cells were transfected with siRNAs against RNF4, USP7, CK2α or GFP and protein levels were assessed by western blotting using the antibodies indicated. These cells were then used for the immunoflourescence microscopy shown in Fig 5.
(TIF)

Figure S4 USP7 overexpression does not affect CK2α silencing. CNE2 cells were treated with siRNA against GFP or CK2α then, 24 hours later, were transfected with a plasmid expressing myc-tagged USP7. 24 hours later, cells were fixed and stained for myc and Ck2α. Similar down-regulation of CK2α was observed in cells with or without myc staining (bottom row).
(TIF)

Figure S5 USP7 is not important for RNF-PML interaction. CNE2 Cells were treated with siRNA against USP7 or GFP in duplicate. 24 hours post transfection, samples were either left untreated or treated with As_2_O_3_ for 8 hours. Following arsenic treatment, samples were fixed and processed for IF microscopy using anit-RNF4 and anti-PML antibodies and counter stained with DAPI.
(TIF)

Figure S6 Individual PML isoforms form nuclear bodies. A. CNE2 cells before (W.T.) and after (shPML) silencing of total PML as shown after staining for total PML (red) and DAPI counterstaining. B. CNE2 cells after silencing of endogenous PML and reconstituting with the indicated single EYFP-tagged PML proteins were fixed, stained with DAPI (blue) and PML antibody (red) and visualized for EYFP (green). Images shown were captured at the same exposure times.
(TIF)
