# Supplementary figures and images for: The Herpesvirus Associated Ubiquitin Specific Protease, USP7, Is a Negative Regulator of PML Proteins and PML Nuclear Bodies
Source: PLoS One. 2011 Jan 31;6(1):e16598. doi: 10.1371/journal.pone.0016598 (PMC3031599; doi:10.1371/journal.pone.0016598)

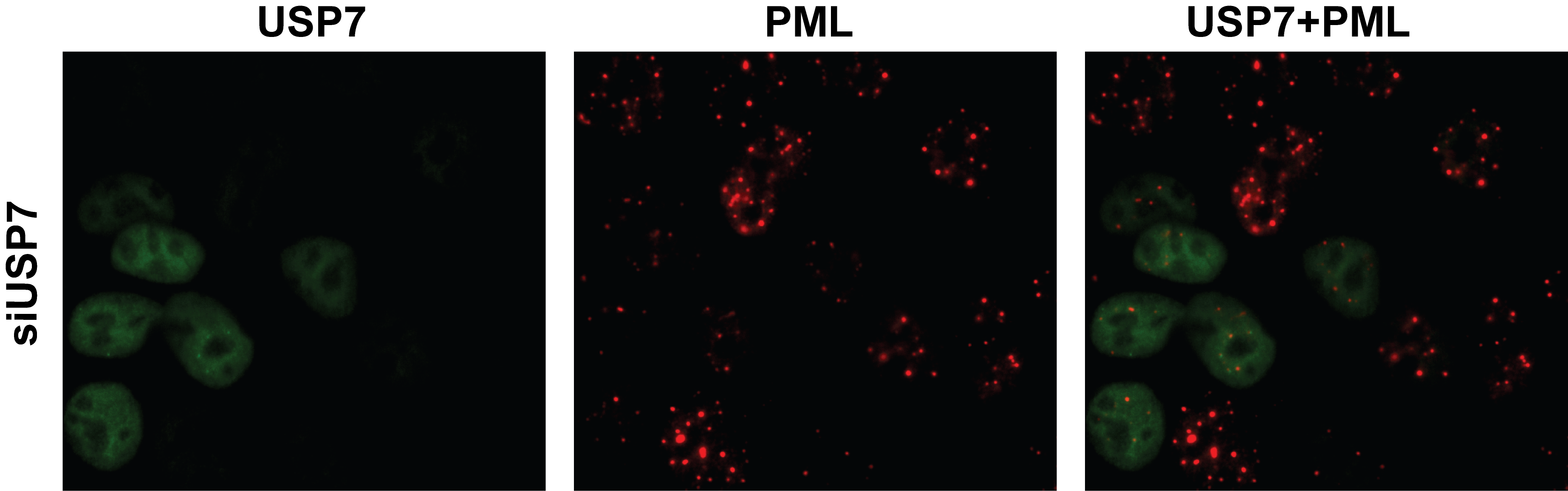

Supplement: Figure S1 — USP7 silencing increases PML staining. CNE2 cells were transfected with siRNA against USP7 and stained for USP7 and PML. An image is shown where a few unsilenced cells remain (green) for direct comparison to the neighbouring cells that are silenced for USP7 expression. (TIF) [file pone.0016598.s001.tif]

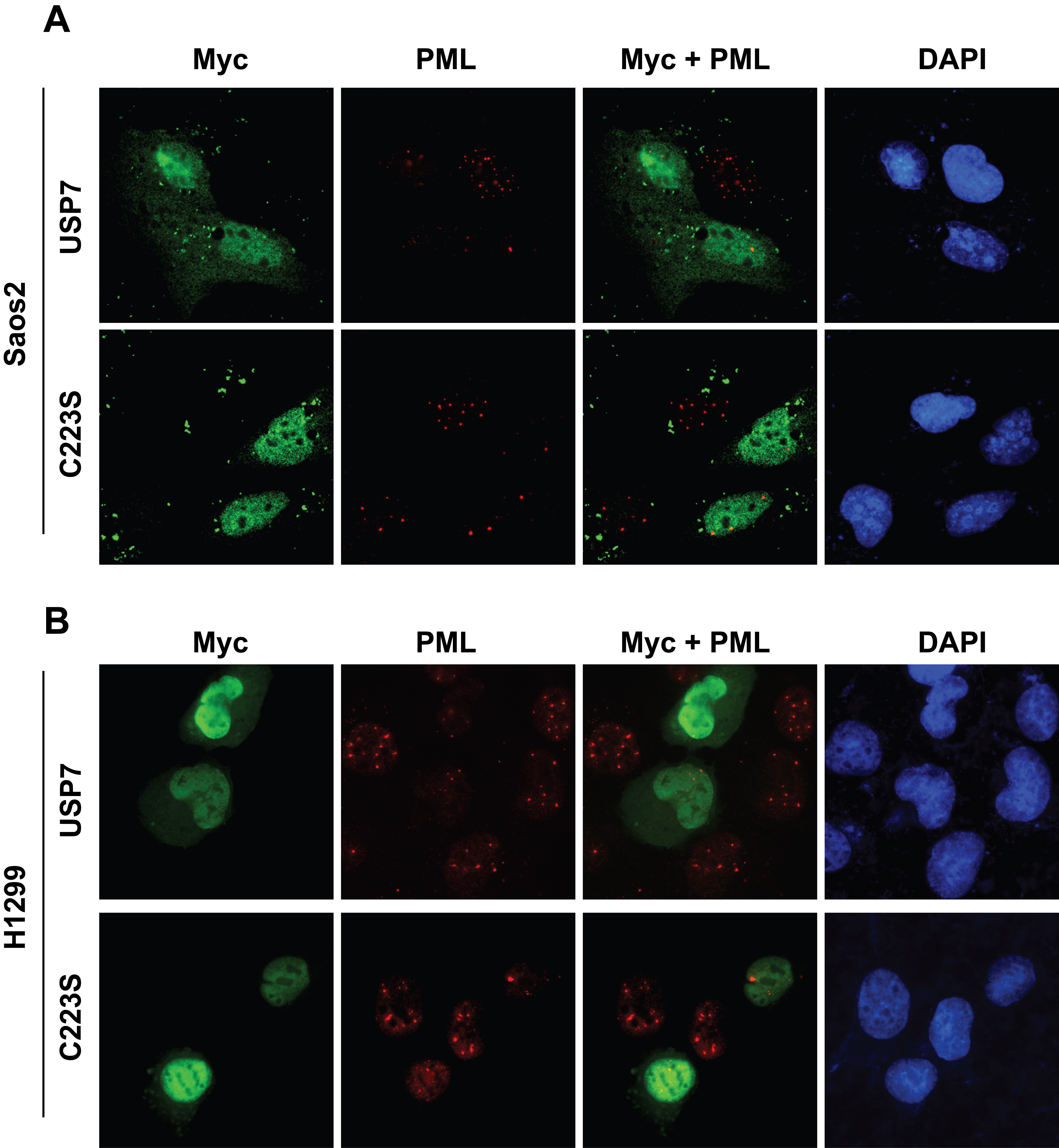

Supplement: Figure S2 — USP7 and C223S overexpression disrupts PML NBs in H1299 and Saos2 cells. Saos 2 (A) or H1299 (B) cells were transfected with a plasmid expressing myc-tagged USP7 or the catalytically inactive USP7 mutant C223S. Cells were fixed and stained for myc and PML as in Figure 1B. (TIF) [file pone.0016598.s002.tif]

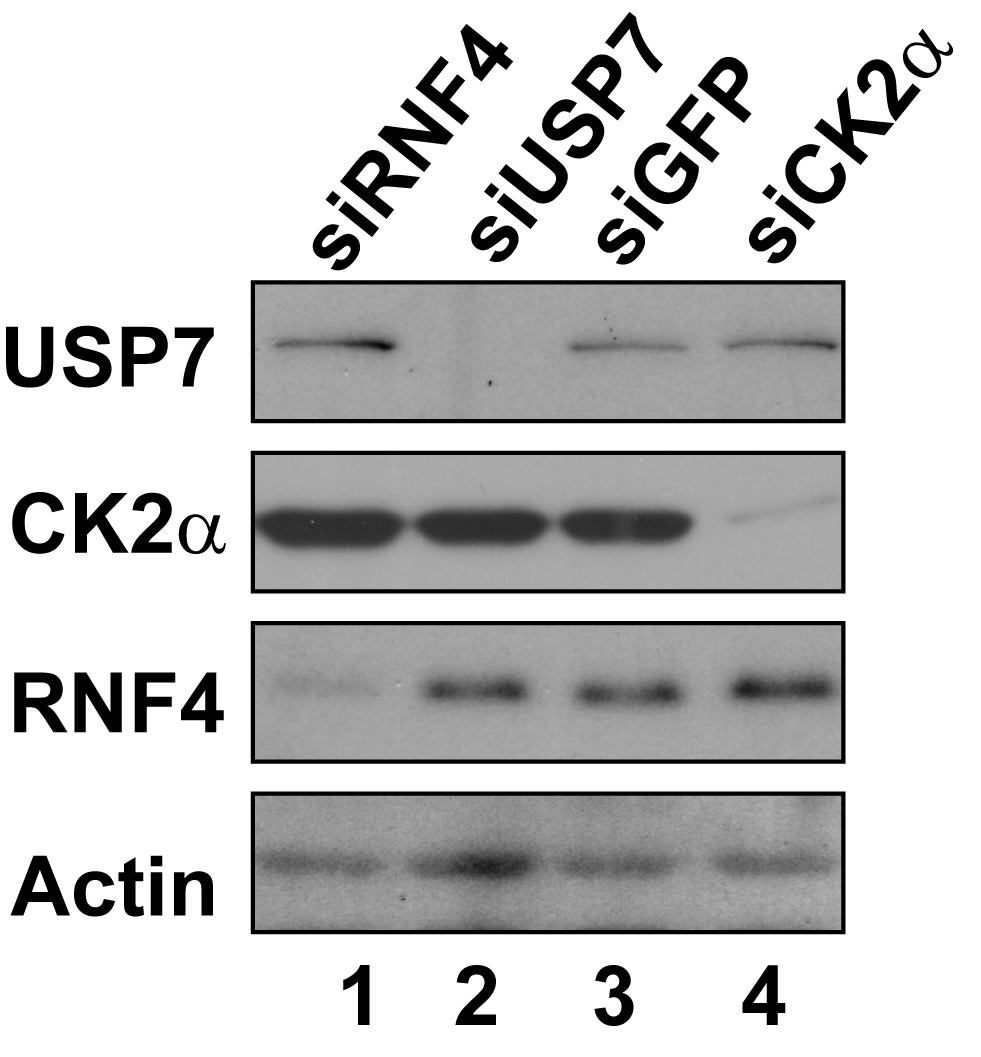

Supplement: Figure S3 — Western blots confirming USP7, RNF4 and CK2α silencing. CNE2 cells were transfected with siRNAs against RNF4, USP7, CK2α or GFP and protein levels were assessed by western blotting using the antibodies indicated. These cells were then used for the immunoflourescence microscopy shown in Fig 5. (TIF) [file pone.0016598.s003.tif]

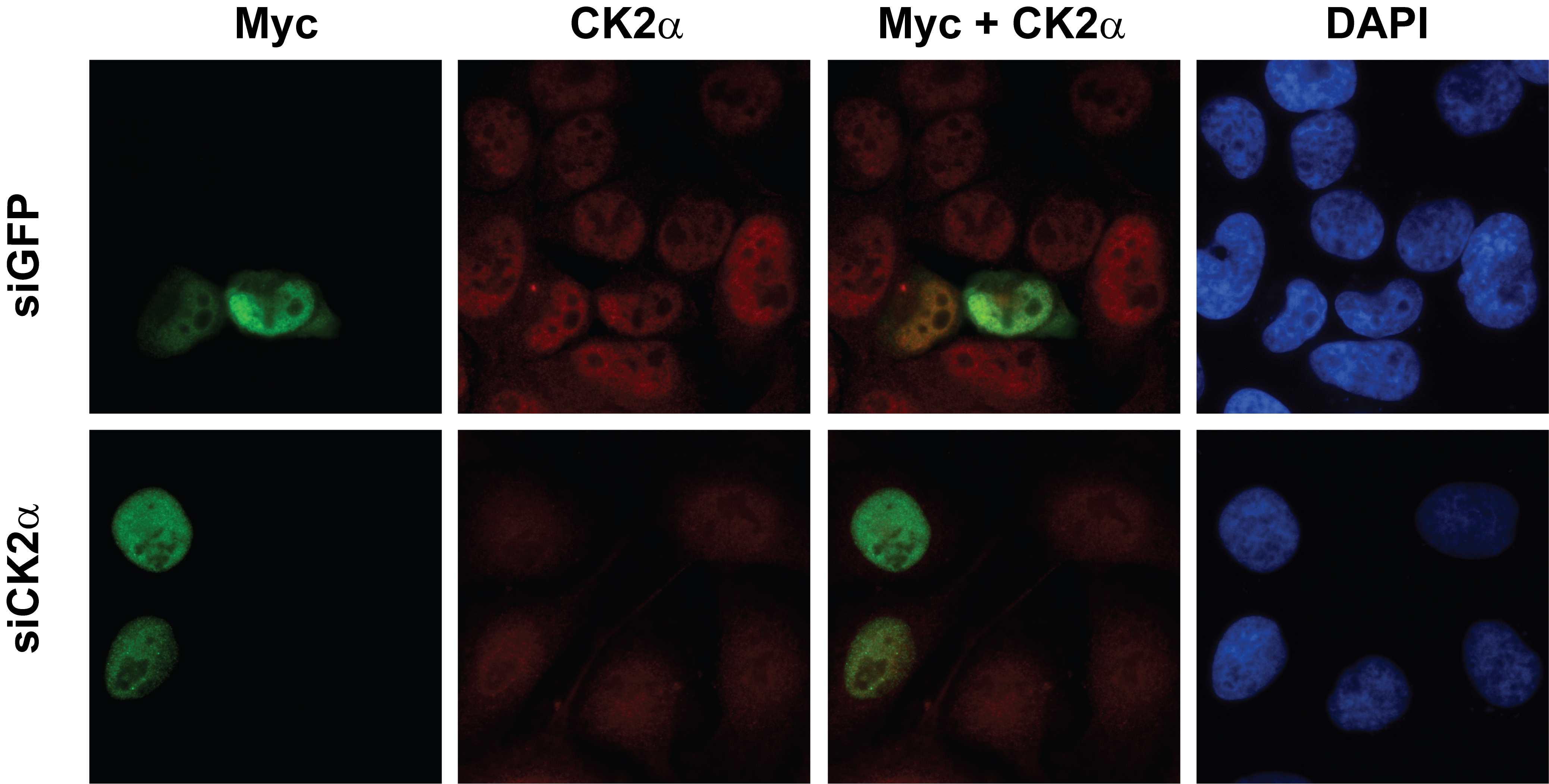

Supplement: Figure S4 — USP7 overexpression does not affect CK2α silencing. CNE2 cells were treated with siRNA against GFP or CK2α then, 24 hours later, were transfected with a plasmid expressing myc-tagged USP7. 24 hours later, cells were fixed and stained for myc and Ck2α. Similar down-regulation of CK2α was observed in cells with or without myc staining (bottom row). (TIF) [file pone.0016598.s004.tif]

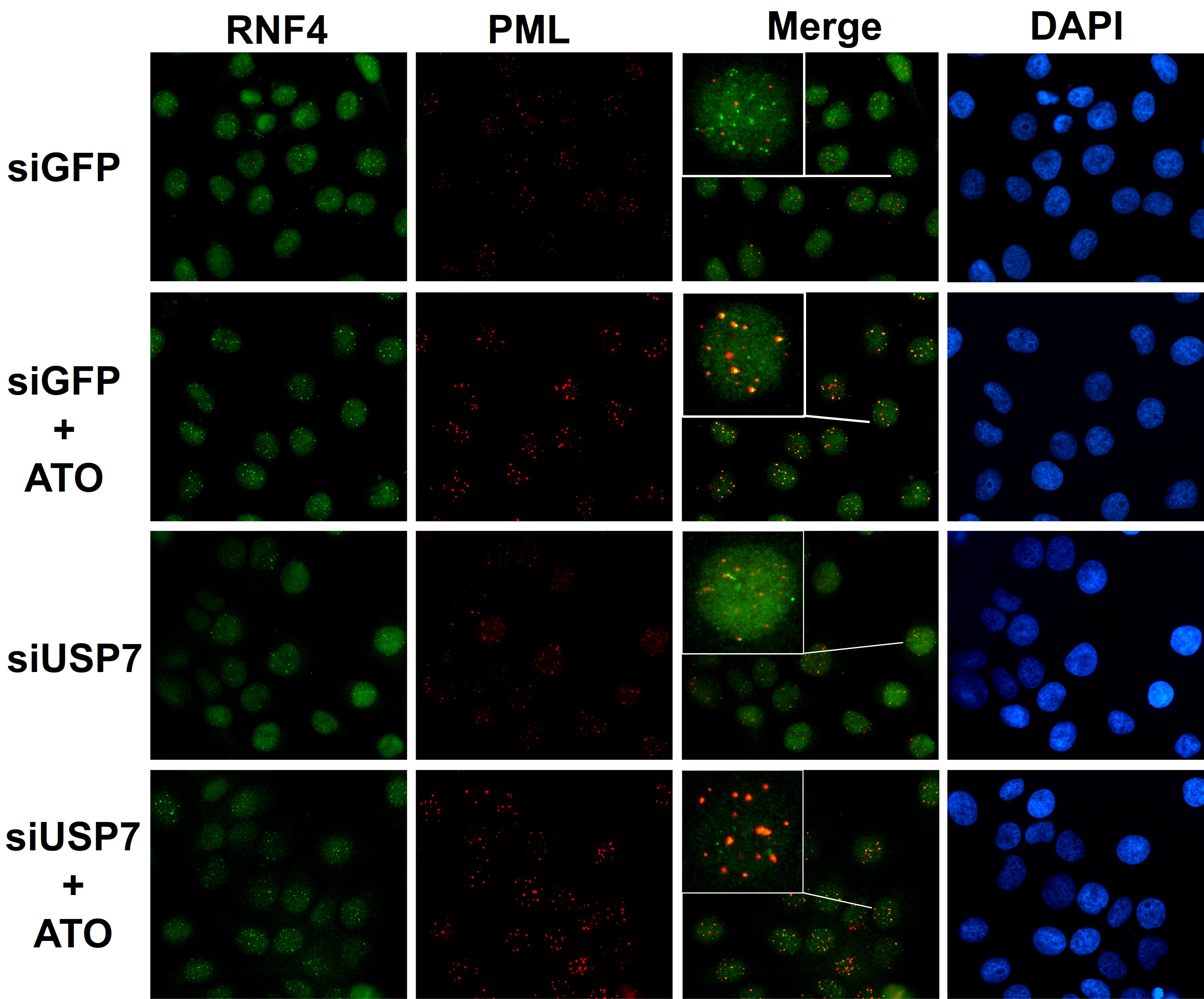

Supplement: Figure S5 — USP7 is not important for RNF-PML interaction. CNE2 Cells were treated with siRNA against USP7 or GFP in duplicate. 24 hours post transfection, samples were either left untreated or treated with As2O3 for 8 hours. Following arsenic treatment, samples were fixed and processed for IF microscopy using anit-RNF4 and anti-PML antibodies and counter stained with DAPI. (TIF) [file pone.0016598.s005.tif]

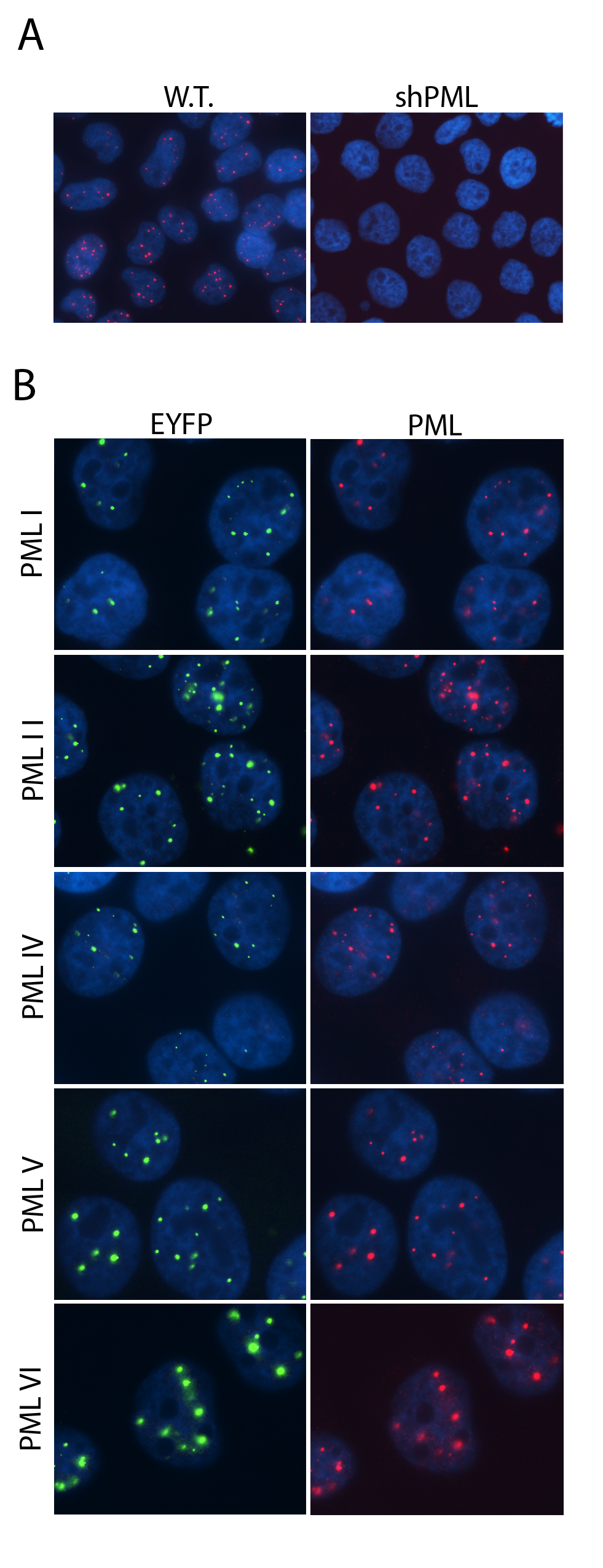

Supplement: Figure S6 — Individual PML isoforms form nuclear bodies. A. CNE2 cells before (W.T.) and after (shPML) silencing of total PML as shown after staining for total PML (red) and DAPI counterstaining. B. CNE2 cells after silencing of endogenous PML and reconstituting with the indicated single EYFP-tagged PML proteins were fixed, stained with DAPI (blue) and PML antibody (red) and visualized for EYFP (green). Images shown were captured at the same exposure times. (TIF) [file pone.0016598.s006.tif]
